# Around the Hospitals

**Published:** 1893-04-29

**Authors:** 


### Around the Hospitals.

Notice to the Managers of Institutions.—Special space is now reserved for the insertion of notices of hospital meetings and festivals. The Editor requests that all notices may reach The Hospital Office, 428, Strand, at least one week before the date of each meeting.

**The Blackburn and East Lancashire Infirmary.**—It was announced in the last annual report of this institution that a Nurses' Home was to be commenced during the year, and we are glad to hear that so much progress has been made that it is hoped the home will be opened about Whitsuntide. The furniture for the new building has been presented by Mr. and Mrs. Henry Harrison, a most generous and helpful gift. The annual report unfortunately shows a decrease in annual subscriptions and donations, but the Board is otherwise to be congratulated on the state of their finance, as they were able, in spite of this falling off, to start the new year with a balance of £109 0s. 11d. Several grants have been made to incurable cases through the Yerburgh Fund (£150 per annum), and Mr. and Mrs. Yerburgh were so much pleased with the satisfactory manner in which the work was carried on, that they placed at the Board's disposal an additional £100. An increase of 72 in the number of patients admitted during 1892 is reported, a decrease of 21 in the number of out-patients. Ninety-three recommendations to convalescent homes were given during the year. The beds allotted to the infirmary at various homes are 11 in number, and are kept fully occupied by patients who have passed through the infirmary, to whom they prove of the greatest benefit. The Board express much regret at the loss by death of four of their number, and also at the enforced resignation of the Rev. A. B. Grosart, D.D., through illness and his leaving the town. Mr. Grosart had been a warm friend to the institution for some five-and-twenty years.

**The Bradford Infirmary.**—When we learn that this institution receives the poor encouragement from the locality of diminishing contributions towards its support, we can but think that want of knowledge of the good work accomplished is the cause. If every inhabitant of Bradford would peruse the admirable report for the present year, we can hardly believe that funds would be still wanting. The number of patients treated has increased, the rate of mortality has diminished. The treatment of patients is furthered by the unusual advantages which the hospital has to offer to convalescents. During the past year 121 persons were sent to convalescent homes, the expenses being borne by the Ollthwaite Fund. To render the nursing in every way efficient, the nursing staff has been added to and ward maids introduced. The operating theatre has been altered to meet modern requirements in the direction of ventilation and purification. It has further been furnished with the electric light, through the generosity of Dr. Grabham. In the out-patients' department renovations and alterations have taken place. The proposals for future improvements include the building of an isolation ward (an obvious necessity) and a new mortuary. The reconstruction of the out-patients' department is also suggested. The hospital has been the recipient of several legacies, and several kind friends have testified their appreciation of the institution by rendering assistance in various ways. The Woodlands Convalescent Home has proved a great blessing to many. Upwards of 600 patients have been received. In connection with the Bradford Infirmary is a useful ladies' committee. Nearly 600 visits were paid to the institution by the members of this body.

